# Prof. Huan-Yong Chen: a leading botanist and taxonomist, one of the pioneers and founders of modern plant taxonomy in China

**DOI:** 10.1007/s13238-016-0311-4

**Published:** 2016-09-27

**Authors:** Rui-Lan Huang

**Affiliations:** South China Botanical Garden, Chinese Academy of Sciences, Guangzhou, 510650 China

**Figure 1:**
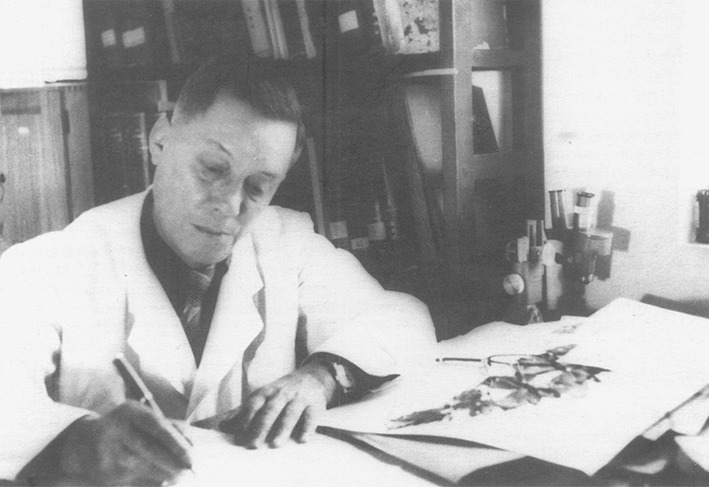
Prof. Huan-Yong Chen at work in 1956 in Guangzhou

**Figure 2:**
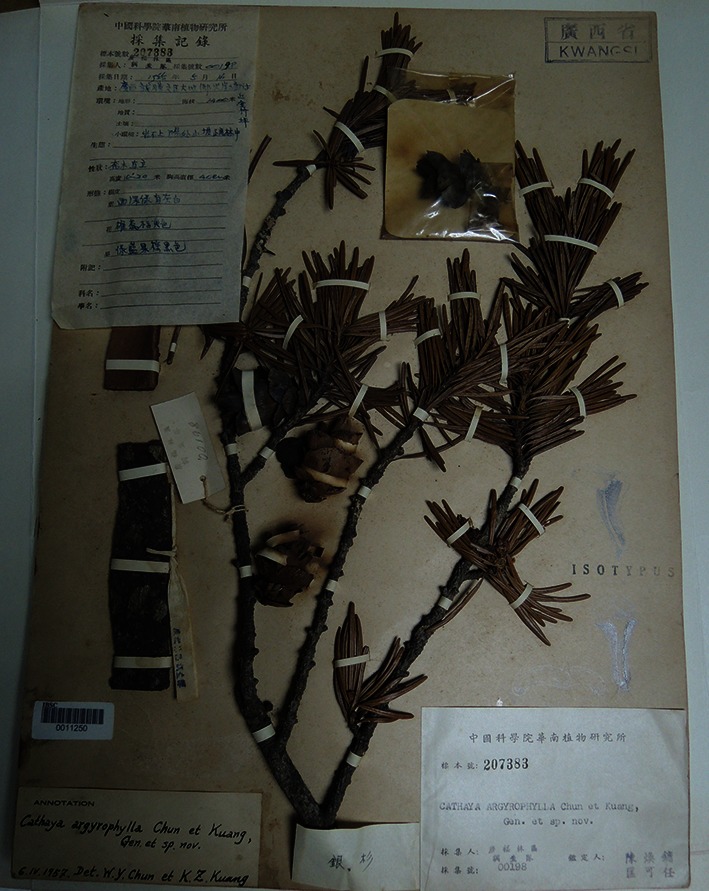
Specimen of *Cathaya argyrophylla* published by Prof. Huan-Yong Chen and Prof. Kuang Ke-Ren

Prof. Huan-Yong Chen (Woon-Young Chun, 陈焕镛, 1890–1971), a leading botanist and taxonomist (Fig. 1), who started a new era for the modern plant science in China and made a great contribution to the plant taxonomy, botany and horticulture of China, especially in Southern China. He was one of the first academicians of the Chinese Academy of Sciences (CAS), a member of the first, second and third China’s National People’s Congress (NPC), one of the first editors of *Flora of Republicae Popularis Sinicae* (FRPS, Flora of China). He was also a leading character in the history of botanical collection by Chinese botanists, and published hundreds of new genera and species, including the famous “living fossil” plant—*Cathaya argyrophylla* Chun et Kuang (Fig. 2).

**Figure 3:**
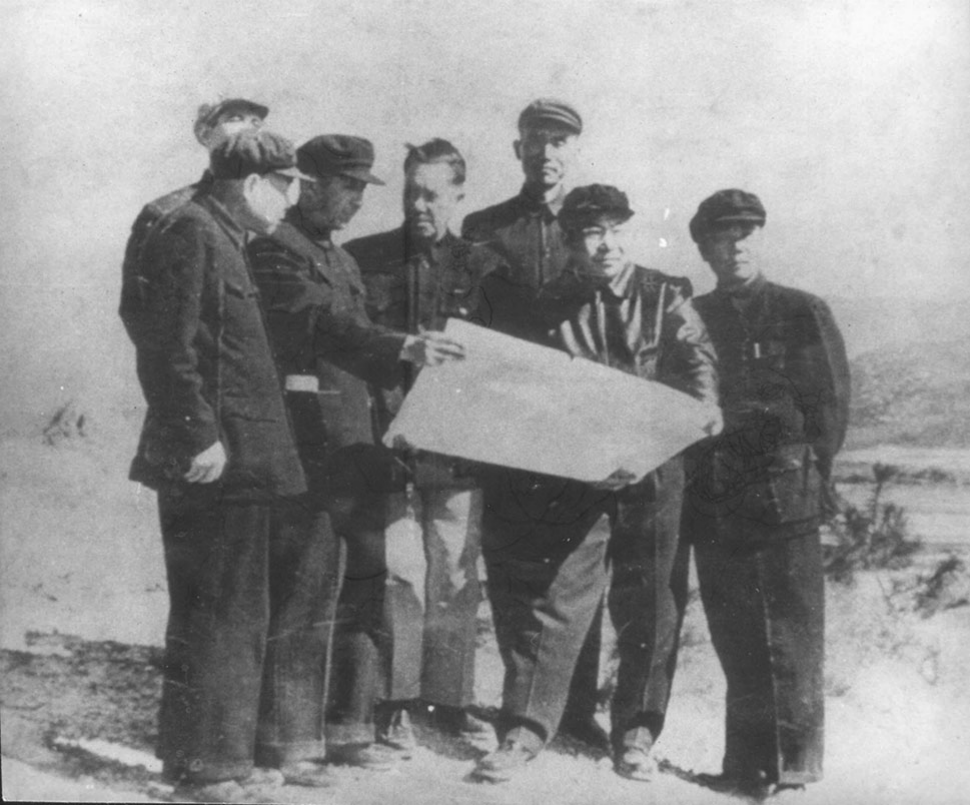
Prof. Huan-Yong Chen (in the *middle*) was discussing the design of the landscape for SCBG with colleagues in 1956

He built up one of the first modern herbariums in China, became the founder and first director of South China Botanical Garden (SCBG), CAS and Guangxi Institute of Plant Research (Fig. 3). Moreover, he established the first national nature reserve Dinghushan Arboretum.

He launched the first English botanic journal *Sunyatsenia* in China, co-founded the Botanical Society of China, and served as the editor and president of *The Chinese Journal of Botany*. In honor of his great contributions, many plants were named after him, such as the genera *Chunia, Chuniophoenix, Chunechites*, and the species *Lindera chunii*, *Neolitsea chui* and *Vernonia chunii,* etc (Chen, 1996). He is one of the most outstanding scientists in the field of botany and taxonomy in early modern China.

**Figure 4:**
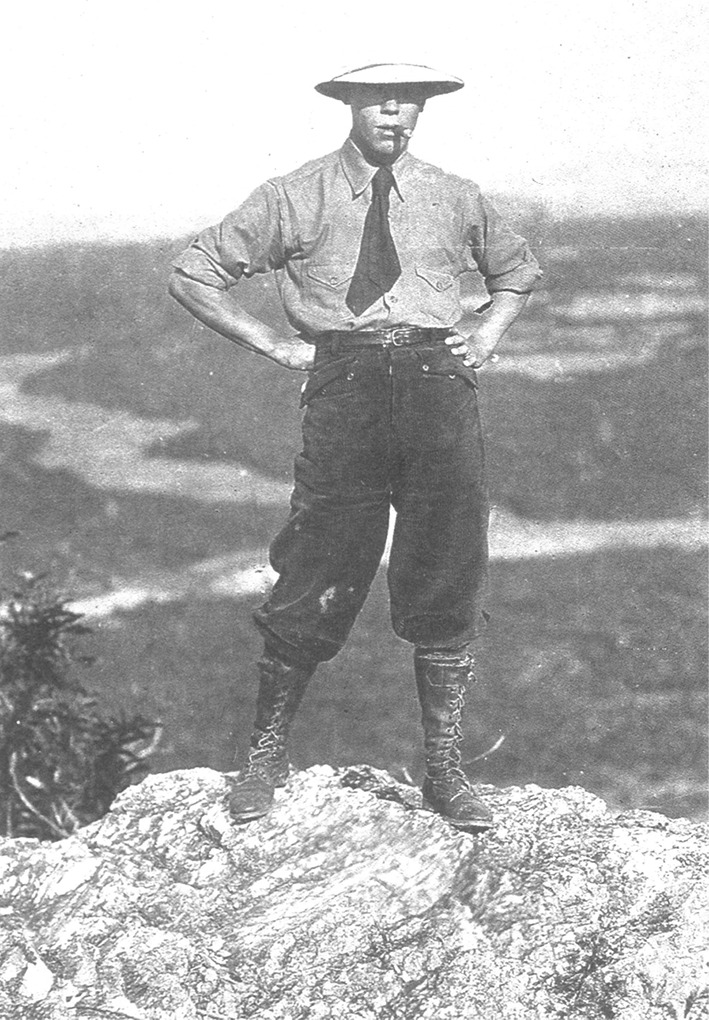
A photo shows Prof. Huan-Yong Chen was collecting plant specimens in Hainan Province in 1919

Born on July 22nd, 1890 in Hong Kong, Prof. Chen grew up in Guangdong and Shanghai, and then traveled to the US in 1905 when he was only 15 years old. He went to Massachusetts Agricultural College in 1909 to study forestry and entomology, and studied at Forestry College of Syracuse University in New York City in 1912. After he received his bachelor’s degree in 1915, he continued his research career at Bussey Institution of Harvard University and obtained a Master’s degree in forestry at the Arnold Arboretum of Harvard University in 1919 (Haas, 1988). He gave up the opportunity for further doctoral studies, instead, he accepted the Sheldon Traveling Fellowship and completed a plant survey and collection in Hainan Island in China (Fig. 4). He spent 9 months and risked his life to explore and collect large number of specimens at Wuzhi Mountain where the flora was barely known before.

**Figure 5:**
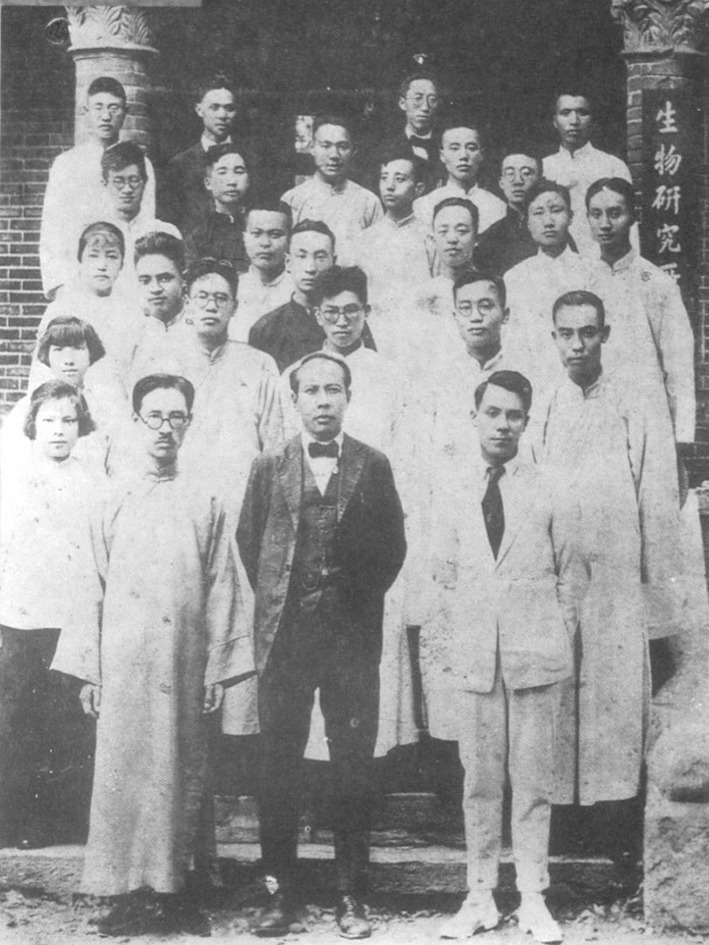
Xian-Su Hu, Bing Zhi and Huan-Yong Chen (From *Left* to *Right* in the first row) together with colleagues and students in the Department of Biology, National Southeast University in 1929

The following year, Huan-Yong Chen was appointed as a professor of forestry at the University of Nanking and started teaching at National Southeast University in 1922, where he worked together with Xian-Su Hu (Hsen-Hsu Hu, 胡先骕), Zhi Bing (秉志) and other renowned biologists in China (Fig. 5). In 1922, together with botanists Chong-shu Qian (Sung-Shu Chien, 钱崇澍) and Ren-chang Qin (Ren-Chang Ching, 秦仁昌), Prof. Chen organized the first Chinese plant collection group to explore the west part of Hubei province, and collected around one thousand specimens. As a talented and hard-working botanist with a broad view and advanced awareness for the development of Chinese botany, Prof. Chen complied the book *Chinese Economic Trees* when he was only 27 years old. It was the first botanical book in English authored by a Chinese botanist, which was translated into Chinese by Feng-Huai Chen (Feng-Hwai Chen, 陈封怀) and published by Commercial Press in 1922.

In 1927, Prof. Huan-Yong Chen was appointed as a professor in Sun Yat-Sen University, and in the following year, he founded and directed a new institute of botany in the university. He helped to establish the professional and qualified herbarium in Southern China in 1928. Furthermore, he made specimens exchanges with more than 60 countries and obtained more than 30,000 foreign specimens to enrich the specimens for herbarium in the 1920s. This herbarium is still considered as one of the top three Herbariums in China till now. In 1929, Prof. Chen was appointed as the first director of Institute of Agriculture and Forestry, which was the predecessor of SCBG. Since then, he devoted his entire life as the director of SCBG and donated hundreds of publications to the library of SCBG. In 1935, he also founded the Economic Institute of Botany in Guangxi University and served as the first director, and he was also involved in the design and construction of the Guilin Botanical Garden (Hu, 2013).

As an educator, Prof. Chen trained numerous famous botanists including Ren-chang Qin, Feng-Huai Chen, Ying Jiang (Ying Tsiang, 蒋英), et al. He also edited and compiled a number of textbooks and reference books for teaching, for example, the Latin version of *Deriration of Genera Names*, the English version of *Chinese Seed Plant Identification*, and books for teaching Latin: *Question from botanical latin* and *Eseential Latin for Chinese taxonomy botany.* In 1930, he established and edited *Sunyatsenia*, the first Chinese professional plant taxonomy journal in English, and till 1948, 7 volumes 26 issues were published (Chen De-Zhao, 1996). This journal gained a great reputation and received awards from home and abroad by its high quality and professional articles.

Prof. Chen became well-known as the leading botanist in South China by both Chinese and foreign scientists in 1930s. He collaborated with Xian-Su Hu and published five volumes of *Icones Plantarum Sincarum* from 1925 to 1932, in which they reviewed all the published illustrations and descriptions of Chinese plants. The scientific researches on the flora in South China gradually expanded under Prof. Chen’s leadership. In 1933, Chong-Shu Qian, Xian-Su Hu and Huan-Yong Chen were invited to found the Botanical Society of China, and in the same year, Prof. Chen was appointed as the editor and academic council member of *The Chinese Journal of Botany*. In 1936, he became the president of *The Chinese Journal of Botany*, after serving as vice president since 1934.

During World War II and the War of Resistance against Japanese Aggression, in order to preserve thousands of precious specimens and literatures, Prof. Chen risked his life to ship them from Guangzhou to Hong Kong. In 1938, with his staff, he managed to keep on doing researches in the Kowlong office until Hongkong was also occupied in 1941. In 1942, he moved the stuff back to Guangzhou in order to continue his research. At that time, he was forced to collaborate with the Puppet Government (Japanese forces), which brought him into trouble in 1945 when the nationalist government accused him for collaborating with the enemy (Hu Zong-Gang, 2013). Although the charges were squashed later, the development and the research work of the institute were badly affected by the lack of research funding and the war.

In spite of those adverse situations, Prof. Chen still chose to stay in Mainland rather than Taiwan when P. R. China was established in 1949. He became the first director of SCBG in 1954. In the same year, he was elected as a member of the first National People’s Congress (NPC), and was subsequently reappointed for second and third NPCs. He was also, a faculty member of Biology and Earth Sciences department in Chinese Academy of Sciences (CAS). In 1955, Prof. Chen was elected as one of the first academicians in CAS for his outstanding contribution in the field of botany and taxonomy, which is the highest honor for a Chinese scientist.

Prof. Chen always admired the beauty of the nature and eagerly participated in conservation since his childhood. He put forwards a joint proposal to set up natural reserves when he attended the first National People’s Congress of China. The proposal was approved in 1958 and he soon established the first national nature reserve Dinghushan Arboretum. In the same year, he described for the first time the new genus *Cathaya* with Ke-Ren Kuang (Ko-Zen Kuang, 匡可任) and published the famous “living fossil” plant—*Cathaya argyrophylla* Chunet Kuang. In addition, he described more than 100 new species and more than 10 new genera including *Tsoongiodendron* (Magnoliaceae) and *Zenia* (Fabaceae) within ten years (Chen, 1996).

In 1959, Prof. Chen was appointed as the Associate Editor-in-Chief of *Flora Republicae Popularis Sinicae* (*FRPS*, *Flora of China*) and later he led a team to complete a survey of the Flora of Guangzhou, which was the first Chinese endemic flora survey completed by Chinese botanists. He also participated in the publication of the Flora in Hainan. In 1963, Prof. Chen was elected as academic chairman of the 30th anniversary conference for Botanical Society of China in Beijing and a member of Academic Committee of Institute of Botany, CAS (Chen De-Zhao, 1996).

Prof. Chen was born mixed-blood parentage, his father was a Chinese Consul in Cuba and his mother was a Spanish Cuban. In addition, he studied and lived in the US for decades, which broadened his international viewpoints and understanding of both eastern and western cultures. He was good at both English and professional Latin, which gave him more opportunities to present and improve the perception of Chinese botanical researches. In fact, before 1949, most of his thesis and academic articles were written in English or Latin.

**Figure 6:**
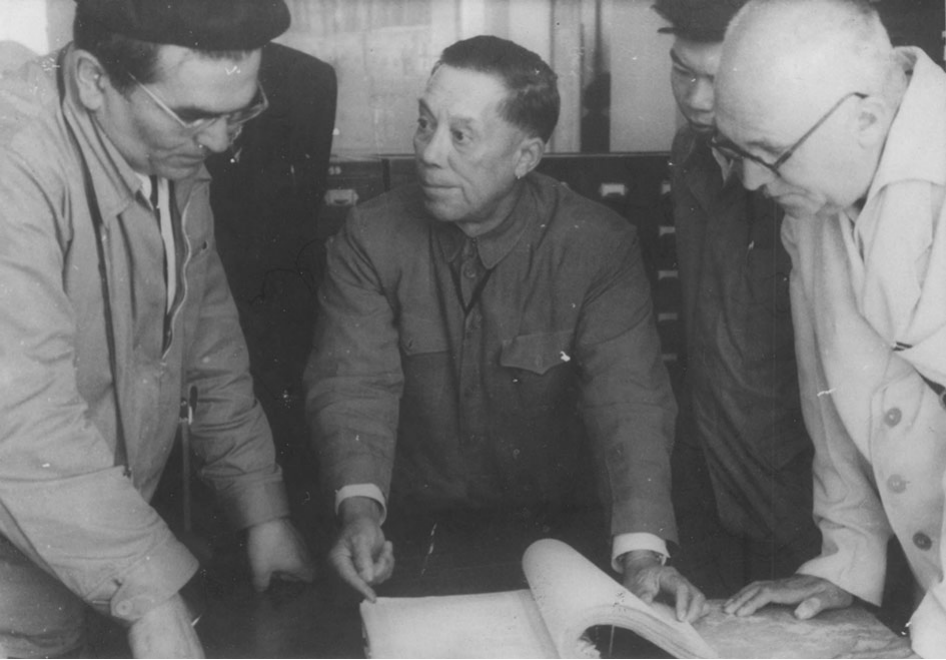
Prof. Huan-Yong Chen worked with Soviet Russia Academicians in 1958

From the 1920s to the 1950s, Prof. Chen attended several important international congresses, including the Third Pan-Pacific Science Conference in 1925, Tokyo, Japan. In 1929, at the Fourth Pan-pacific Science Congress in Java, Indonesia, he presented a poster about the Flora of Guangdong. In 1930, at the Fifth International Botanical Congress in Cambridge, England, a symposium on the Flora of China was held for the first time in the history. The Chinese botanists attended this symposium and in the address to the symposium, Prof. Chen overviewed the development of botany in China (Hu, 2013). In 1931, Prof. Chen attended the Fifth World Plant Conference as the Chinese team leader. Xian-Su Hu and he were elected as the board members of *Species Muscorum Frondosorum* (International Code of Botanical Nomenclature), which was a significant milestone of a worldwide appreciation of Chinese botanists. In 1936, Prof. Chen was awarded as Honorary Vice President of British Gladiolus Society, and appointed as a member of Massachusetts Horticulture Society (MassHort, US). In 1951, at the symposium on Origin and distribution of cultivated plants of South Asia held in New Delhi, India, Prof. Chen presented his research work as the head of the delegation of CAS. He also visited several Plant Research Centers on behalf of CAS and helped identify plant specimens in Leningrad (Saint Petersburg), former Soviet Union in 1958 (Fig. 6) (Chen, 1958–1963).

All through his life, Prof. Chen kept a good relationship with top botanists all over the world. The former director of Arnold Arboretum, Professor C. S. Sargent, supported him during his early career, and the famous botanist Dr. Elmer Drew Merrill (1876–1956) kept a life-long friendship with him ever since they meet in 1920. Dr. Merrill donated a lot of precious publications to the library and specimens to the herbarium, and also wrote an article for the first issue of the journal *Sunyatsenia*.

In addition to his botanic research, Prof. Chen was also interested in literature. He read lots of European and American novels from the library during his study at Harvard University and some of his poems were published in the Hong Kong newspaper *South China Morning Post* (Chen, 1996).

Prof. Chen passed away in 1971, and left countless treasures for the next generations. In 1978, a memorial meeting was held by Guangdong Academy of Sciences. Many government officials and scientists attended this meeting to greet Prof. Chen’s outstanding contributions to the development of Chinese botanical sciences. In 1996 and 2006 respectively, his bronze statues were built in the Herbarium and the garden of SCBG. And the internal book of *Memorial collections of Huan-Yong Chen* was published in 1996 to memorize all his endeavors to the development of SCBG.

As one of the most outstanding botanists in 20th century and one of the pioneers and founders of modern plant taxonomy in China, with a strong determination to establish the modern Chinese botanical research, Prof. Chen considered the development of Chinese botanical science as his life-long mission. He was blessed to do what he loved, and made his dream come true-to set up China’s own botanical research, Herbarium, botanical library, Chinese flora, botanical gardens/institutions and natural reserves, which were deeply respected all over the world.
