# Correction to: Prof. Huan-Yong Chen: a leading botanist and taxonomist, one of the pioneers and founders of modern plant taxonomy in China

**DOI:** 10.1007/s13238-018-0569-9

**Published:** 2018-07-24

**Authors:** Rui-Lan Huang

**Affiliations:** South China Botanical Garden, Chinese Academy of Sciences, Guangzhou, 510650 China

## Correction to: Protein Cell (2016) 7(11):773–776 10.1007/s13238-016-0311-4

In the original publication, there are some incorrect information.

The correct information is provided in this Correction:

The year in caption of Figure 5, should be “1925” not “1929”.

The sixth line of third paragraph in first page the Latin name “Neolitsea chui” should be “Neolitsea chuii”.

The year in tenth line of third full paragraph of page 775, should be “1935” not “1931”. In the twelfth line of same paragraph, “fifth” should be read as “sixth”.

